# *Tetraselmis chuii* Edible Microalga as a New Source of Neuroprotective Compounds Obtained Using Fast Biosolvent Extraction

**DOI:** 10.3390/ijms25073897

**Published:** 2024-03-31

**Authors:** Melis Cokdinleyen, Gerardo Alvarez-Rivera, Jose Luis González Tejera, José A. Mendiola, Alberto Valdés, Huseyin Kara, Elena Ibáñez, Alejandro Cifuentes

**Affiliations:** 1 Laboratory of Foodomics, Institute of Food Science Research, CIAL, CSIC—UAM, Nicolas Cabrera 9, 28049 Madrid, Spainelena.ibanez@csic.es (E.I.); 2 Faculty of Science, Department of Chemistry, Selçuk University, Ardıçlı, İsmetpaşa Cad, Selçuklu, 42250 Konya, Turkey

**Keywords:** *Tetraselmis chuii*, green extraction, QTOF, carotenoids, polyunsaturated fatty acids, neuroprotective effect, pressurized liquid extraction

## Abstract

*Tetraselmis chuii* is an EFSA-approved novel food and dietary supplement with increasing use in nutraceutical production worldwide. This study investigated the neuroprotective potential of bioactive compounds extracted from *T. chuii* using green biobased solvents (ethyl acetate, AcOEt, and cyclopentyl methyl ether, CPME) under pressurized liquid extraction (PLE) conditions and supercritical fluid extraction (SFE). Response surface optimization was used to study the effect of temperature and solvent composition on the neuroprotective properties of the PLE extracts, including anticholinergic activity, reactive oxygen/nitrogen species (ROS/RNS) scavenging capacity, and anti-inflammatory activity. Optimized extraction conditions of 40 °C and 34.9% AcOEt in CPME resulted in extracts with high anticholinergic and ROS/RNS scavenging capacity, while operation at 180 °C and 54.1% AcOEt in CPME yielded extracts with potent anti-inflammatory properties using only 20 min. Chemical characterization revealed the presence of carotenoids (neoxanthin, violaxanthin, zeaxanthin, α- and β-carotene) known for their anti-cholinesterase, antioxidant, and anti-inflammatory potential. The extracts also exhibited high levels of omega-3 polyunsaturated fatty acids (PUFAs) with a favorable ω-3/ω-6 ratio (>7), contributing to their neuroprotective and anti-inflammatory effects. Furthermore, the extracts were found to be safe to use, as cytotoxicity assays showed no observed toxicity in HK-2 and THP-1 cell lines at or below a concentration of 40 μg mL^−1^. These results highlight the neuroprotective potential of *Tetraselmis chuii* extracts, making them valuable in the field of nutraceutical production and emphasize the interest of studying new green solvents as alternatives to conventional toxic solvents.

## 1. Introduction

Due to the increase in life expectancy in the XXI century, neurodegenerative diseases are predicted to be the second most common cause of death among the elderly by 2040s [1]. More than 600 neurological disorders have been identified to date, and Alzheimer’s disease is the most common, accounting for 60–70% of all dementia cases (AD) [2].

Although the pathogenesis of AD is multifactorial, it is mainly based on extracellular β-amyloid plaque deposition and intraneuronal aggregation of neurofibrillary tangles (NFT) [3]. Aβ clustering around neurons has a toxic effect and sensitizes neurons, triggering the production of free radicals [4,5]. As a result, reactive oxygen and nitrogen species (ROS and RNS) cause neuroinflammation, oxidative/nitrosative damage, microtubule disassembly, synaptic loss, neuronal cell death, and axonal degeneration [6]. Since oxidative stress plays an important role in the initiation and progression of neurodegeneration, it is important in the development of neuroprotective compounds [7]. Another common biochemical change in AD patients is the decrease in acetylcholine (ACh) and butyrylcholine (BuCh) levels in the hippocampus and cortex of the brain. Therefore, inhibition of acetylcholinesterase (AChE) and butyrylcholinesterase enzymes (BuChE) responsible for the hydrolysis of ACh and BuCh has also become a treatment option for AD [8].

Over the last few years, microalgae have gained increasing attention in the pharmaceutical and food industries as they are recognized as great potential sources of bioactive compounds and functional ingredients [9]. *Tetraselmis chuii* [10] is a single-celled green microalga belonging to the Prasinophyceae class. *T. chuii*, which does not produce toxins, is harmless to other marine species, and has been approved as a ‘novel food’ by the American Food and Drug Administration (FDA) and the European Food Safety Authority (EFSA) [11,12]. This microalga is a high nutritional source, since it contains all essential amino acids, long-chain polyunsaturated fatty acids (PUFAs), and fat-soluble carotenoids [13]. This composition makes *T. chuii* an excellent source of bioactives for the food, pharmaceutical, and cosmetic industries. In this sense, *T. chuii* has been studied with regard to its antioxidant and anti-inflammatory properties. For instance, *T. chuii* extracts characterized by a substantial phenolic and rich lipidic contents demonstrated high antioxidant efficacy in terms of DPPH radical scavenging power and ORAC activity [14]. Other studies of *T. chuii* extract display potent inhibitory activity against nitric oxide (NO) in macrophage cells, with this effect being ascribed to the presence of specific polar lipids, specifically galactolipids and betaine lipids [15]. Studies investigating the supplementation with dried extracts from *T. chuii*, such as TetraSOD^®^, at varying doses, have shown positive outcomes in reducing oxidative stress and inflammation, enhancing the liver’s innate antioxidant defense mechanisms; regulating oxidative stress and inflammatory markers in the bloodstream; and modulating genes associated with antioxidant, anti-inflammatory, and immunomodulatory pathways in various organs [13,14,15,16,17]. However, no previous work has been conducted on the potential neuroprotective activity of *T. chuii* extracts.

Conventional methods to recover bioactive compounds from complex natural sources are still widespread in the literature, but they require the use of high volumes of organic solvents (e.g., dichloromethane, chloroform, methanol) and long extraction times, precluding its sustainability. Nowadays, these classical approaches are being replaced by new green extraction processes based on compressed fluids technologies (supercritical fluid extraction (SFE), pressurized liquid extraction (PLE)) or by non-thermal and low-pressure techniques, such as pulsed electric fields (PEF), that contribute to increase extraction efficiency, shortening processing time, and reducing environmental damage caused by the use of large amounts of toxic solvents [18]. In this sense, SFE [19,20] and PEF [21] have been employed to isolate pigments and polyphenols from *T. chuii*, showing antioxidant activity.

PLE is a fast and efficient extraction method that uses high temperatures and pressures, keeping the solvent in liquid state. The so called ‘generally recognized as safe solvents’ (GRAS) such as ethanol, water, or ethyl acetate can be used in green extraction approaches, reducing the toxicity and the environmental impact of extractions. Among these green solvents, cyclopentyl methyl ether (CPME) is considered as one of the few green non-polar solvents, and it is gaining attention in the field of extractions and biorefineries [22]. In fact, it has demonstrated its potential as a green non-polar solvent to obtain lipids from microalgae [23], as well as ethyl acetate (AcOET) [24]. Both are well-recognized bio-based solvents that can be applied within a Green Chemistry framework. The high pressures and temperatures in PLE procedures modify the transport properties of the solvents by increasing solubility and decreasing viscosity, which result in higher mass transfer rates and penetration capacity into the matrix, and consequently higher extraction efficiency is achieved [25,26,27]. The use of compressed liquid-based extraction techniques to recover bioactive compounds from various microalgae species is a hot research topic, as recently described by Gallego et al. [27], who obtained PLE carotenoid-rich extracts with cholinesterase inhibitory potential and high antioxidant and anti-inflammatory capacity from different microalgae species. On the other hand, no reports can be found in the literature on the use of PLE, together with biobased solvents, to isolate potential bioactive compounds from *T. chuii*. 

Therefore, the primary objective of the current research was to explore the potential of *T. chuii* to provide neuroprotection through an optimized pressurized liquid extraction (PLE) procedure. This procedure involved the use of solvents that are generally recognized as safe (GRAS). In addition, the study aimed to determine whether the bioactivity of the extracts obtained was affected by a biorefinery process that combined supercritical fluid extraction (SFE) and PLE. The neuroprotective properties of the extracts were evaluated by a series of in vitro assays, including the capacity to scavenge reactive oxygen/nitrogen species (ROS/RNS) and inhibit key enzymes such as acetylcholinesterase (AChE), butyrylcholinesterase (BuChE), and lipoxygenase (LOX). These assays shed light on the ability of the extracts to counteract oxidative stress and inflammatory processes, which are key factors in neurodegenerative diseases. In addition, the toxicity of the optimized extracts was assessed in cell lines as a first step to in vivo studies. The bioactive compounds obtained from *T. chuii* was analyzed using a multi-analytical platform that employs chromatographic techniques (GC and LC) coupled with high-resolution mass spectrometry. This analysis provides valuable insights into the potential mechanisms of action of these compounds.

## 2. Results and Discussion

The proposed experimental design to optimize the bioactive potential of *T. chuii* extracts was based on a response surface methodology considering two factors: extraction solvent (0–100% ETAC–CPME) and extraction temperature (40, 110, and 180 °C). ETAC and CPME were selected based on their low toxicity, biodegradability, and high extraction capacity. The rest of the extraction variables (a static extraction time of 20 min, an extraction pressure of 10.3 MPa, and one extraction cycle) remained constant, based on the experience from previous works by our research group [28].

**Figure 1:**
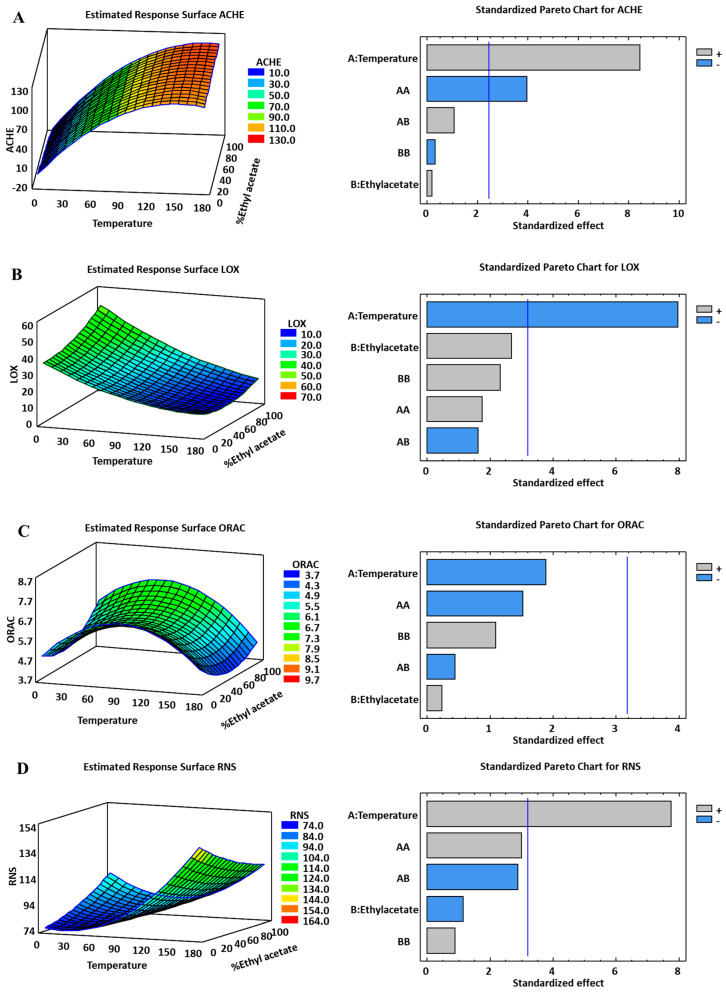
Standardized Pareto charts and Response Surfaces for the six response variables studied in the experimental design for the PLE of *Tetraselmis chuii*. (**A**) AChE: acetylcholinesterase IC50, (**B**) LOX: lipoxygenase, (**C**) ORAC: oxygen radical absorbance capacity, (**D**) RNS: reactive nitrogen species. In the response surfaces, higher activities are shown in color scale from blue (lower activity) to red (higher activity). In Pareto Charts gray and blue bars show positive or negative effects, respectively; the vertical blue bar indicates significance at *p* > 0.05.

The three-level factorial design allowed us to assess simultaneously and independently the effect of the extraction parameters in six different response variables, i.e., the extraction yield, TPC, AChE, LOX, ORAC, and RNS. The experimental matrix and the results obtained for each extraction condition are shown in Table 1. The response surfaces of each response variable and their corresponding standardized Pareto charts are shown in Figure 1.

The strong influence of temperature found for all response variables was expected since it is an important parameter in PLE extraction. Temperature affects the molecular diffusion and viscosity of the solvent. The use of higher temperatures in extraction processes can increase the solubility and recovery of compounds from matrices. However, it is critical to determine the optimal extraction temperature for each specific material, as higher temperatures can also lead to degradation of thermosensitive compounds [26]. Nevertheless, in PLE, the short extraction times and the absence of oxygen reduce the rates of thermal degradation. Except for reactive oxygen species (ROS-ORAC), an increase in temperature leads to higher values for extraction yield, total phenolic content (TPC), acetylcholinesterase (AChE) activity, and reactive nitrogen species (RNS) scavenging capacity (Figure 1). On the other hand, lipoxygenase (LOX) activity values decrease with increasing temperature. 

On the other hand, the effect of solvent composition is not uniform across all response variables. For example, higher yields are obtained at high temperatures, regardless of the solvent used. Interestingly, the Pareto plot shows that neither the solvent nor its quadratic term had a significant effect on the extraction yield. In contrast, a higher ORAC response was observed when pure solvents were used, while the mixture of solvents provided a lower ORAC activity. However, within the experimental range studied, no significant effect of temperature or solvent composition could be attributed to explain the variations in ORAC values. The effect of experimental factors on the response variables as determined by the optimization models is discussed in detail in the following section.

### 2.1. In Vitro Neuroprotective Potential Optimization

#### 2.1.1. Anticholinergic Activity In Vitro Assays 

Extracts obtained at low temperature (40 °C) showed the highest AChE inhibitory capacity, that is, lower IC50 values (Figure 1). These conditions increased the AChE inhibition capacity compared to the high temperature extracts (*p* < 0.05) (Table 1). This effect could be related to the thermosensitivity of compounds responsible for AChE inhibition. In addition, the low temperature extracts significantly increased the BuChE inhibition capacity (Table 2). As a reference inhibitor, galantamine, a drug used in Alzheimer’s disease treatment, provided value for the AChE enzyme equal to 1.395 ± 0.193 µg mL^−1^. There are limited studies on the AChE inhibitory potential of algae extracts; the results obtained yielded IC50 values that varied significantly depending on the extraction method and algae species [28]. Several studies suggest that AChE inhibitory activity of microalgae extract is strongly associated with the content of phytosterol, polyphenols, terpenoids, chlorophylls, and carotenoids [29,30,31,32,33]. For example, Gallego et al. [27] obtained carotenoid-rich extracts by PLE from four different algae species, namely, *Haematococcus pluvialis*, *Nannochloropsis oceanica*, *Tisochrysis lutea,* and *Porphyridium cruentum*, showing IC50 values of 87.14, 66.29, 47.17, and 89.21 μg mL^−1^, respectively. A significant difference was observed between the AChE inhibition effect of phytosterol-rich extracts from *Phormidium autumnale* cyanobacteria using green extraction techniques and a traditional extraction method, with an IC50 value of more than twenty times lower [34]. The PLE procedure in these studies shows that the use of green extraction techniques in the recovery of bioactive compounds with anticholinergic potential is more effective when compared to studies with traditional extraction methods. 

The results obtained in our study are comparable to those reported by Gallego et al. [27]. Our extracts showed moderate AChE inhibitory activity, according to the general classification criteria reported for this bioactivity [35,36], where moderate activity is typically defined by IC50 values between 20 and 200 µg mL^−1^. In Figure 1, it can be observed that extracts obtained at lower temperatures showed higher anticholinergic activities, as evidenced by lower IC50 values, regardless of the solvent used. Specifically, IC50 values of 41 µg mL^−1^ were obtained with pure chloroform-methanol (CPME) and pure ethyl acetate (ETAC), while a value of 52 µg mL^−1^ was obtained with the solvent mixture. However, as the temperature increased, the IC50 values also increased, indicating a decrease in anti-AChE activity, regardless of the solvent composition.

These results highlight the influence of temperature on the anticholinergic activity of the extracts. Lower temperatures seem to favor a higher inhibitory activity against AChE, as reflected by the lower IC50 values. On the other hand, increasing temperatures seem to decrease the anti-AChE activity, regardless of the solvent used or its composition.

These results provide important insights into the optimal extraction conditions for obtaining extracts with desirable anticholinergic properties. The choice of lower temperatures during the extraction process may enhance the potential therapeutic value of the extracts in terms of their ability to inhibit AChE, a key enzyme involved in neurodegenerative diseases such as Alzheimer’s disease. 

In particular, these results contribute to the understanding of the relationship between extraction conditions and bioactivity of *T. chuii* extracts, which has never been studied to date.

#### 2.1.2. LOX In Vitro Assay 

Neurodegenerative diseases, including Alzheimer’s disease, are often associated with inflammation. Therefore, the neuro-inflammatory protective effect of the extracts was evaluated in vitro through the inhibition of the enzyme lipoxygenase (LOX). Table 1 and Figure 1 show a clear linear correlation between extraction temperature and LOX inhibitory capacity of the extracts. Specifically, high extraction temperatures (180 °C) resulted in the highest inhibitory effect, as indicated by the lowest IC50 values. Furthermore, regardless of the extraction temperature, all extracts showed a higher inhibitory capacity compared to the positive control (*p* < 0.05), which in this case was quercetin (Table 1). The IC50 values obtained in our study, ranging from 10 to 40 μg mL^−1^, were similar to those reported for other algal extracts [27]. Notably, these IC50 values were lower than those reported for well-studied potential sources of antioxidant compounds such as orange or olive leaf extracts [37,38]. 

The results suggest that extracts obtained using ETAC and/or CPME under pressurized conditions have significant anti-inflammatory properties. In particular, both ETAC and CPME solvents, when used at high temperatures, were effective in producing extracts with strong anti-inflammatory properties. Thus, it can be concluded that the use of these solvents in the pressure extraction process can effectively preserve and extract bioactive compounds with significant anti-inflammatory properties from the *T. chuii* microalga.

Our study highlights the potential of *T. chuii* extracts in reducing neuroinflammation, a crucial factor in the development of neurodegenerative diseases. The extracts’ ability to inhibit LOX, an enzyme involved in producing pro-inflammatory agents, highlights their anti-inflammatory properties and potential as therapeutic remedies for conditions characterized by neuroinflammation. These extracts demonstrate significant LOX inhibitory efficacy, surpassing that of the standard control, and exhibit comparable or even superior anti-inflammatory potential to extracts from other natural sources.

#### 2.1.3. Antioxidant In Vitro Assays 

The antioxidant potential of PLE *Tetraselmis chuii* extracts was evaluated by measuring their ability to inhibit reactive oxygen species (ROS) using the oxygen radical absorbance capacity (ORAC) test, as well as their ability to scavenge reactive nitrogen species (RNS). Both ROS and RNS play important roles in redox stress signaling, and their levels are regulated by antioxidants.

In the context of ROS-ORAC antioxidant analysis, the extracts obtained at 180 °C showed significantly lower IC50 values, indicating a higher level of antioxidant activity, as shown in Table 1. Despite exploring various experimental conditions, no statistically significant correlation between extraction temperature, solvent composition, and ROS activity was found. However, it is important to note that the extracts obtained at 180 °C using the ETAC:CPME (1:1) solvent mixture showed antioxidant activity similar to that of ascorbic acid, a well-known antioxidant compound. These findings suggest that the PLE extracts obtained at high temperatures using the ETAC:CPME solvent mixture have potent antioxidant properties.

In contrast, the analysis of reactive nitrogen species (RNS) activity revealed that the extracts obtained at 40 °C exhibited significantly higher activity (*p* < 0.05) (Figure 1). Interestingly, all extracts obtained in this study demonstrated approximately nine to ten times higher RNS activity compared to the positive control, ascorbic acid (Table 1). It is worth noting that the effectiveness of different solvents on RNS activity varied depending on the extraction temperature. Specifically, at lower temperatures, the RNS activity of pure chloroform-petroleum ether (CPME) was superior (lower IC50 values), while at 180 °C, superior results were achieved with pure ethyl acetate (ETAC).

These results indicate that the pressurized liquid extracts of *T. chuii* possess significant antioxidant capacity, as demonstrated by their ability to eliminate reactive oxygen species (ROS-ORAC) and RNS. The extracts obtained at higher temperatures showed enhanced ROS activity, while those obtained at lower temperatures showed superior RNS activity. The choice of solvent also influenced the antioxidant performance, with pure CPME and pure ETAC showing favorable results under certain conditions.

Overall, the PLE extracts obtained in this investigation exhibited remarkable antioxidant potential. These results underline the suitability of the PLE method, especially when using optimized extraction temperatures and solvent compositions, for obtaining extracts with high antioxidant capacities. Such extracts could have significant implications for the prevention and treatment of disorders associated with oxidative stress, including neurodegenerative diseases.

The results of previous studies indicated that the extracts obtained from different algae species such as carotenoid in particular and phenolic compounds are directly correlated with their antioxidant capacities [27,39,40,41]. Carotenoid molecules such as β-carotene, lutein, violaxanthin, and neoxanthin are very effective in preventing the oxidation of polyunsaturated fatty acids and scavenging oxygen radicals with their highly conjugated system. Non-polar carotenoids can be dissolved in the oil phase to prevent lipid oxidation, whereas less solubility of more polar phenolic compounds in the oil phase probably affects their antioxidant activity [42]. On the other hand, Banskota et al. [14,42] revealed a statistically significant correlation (*p* < 0.05) between the total lipid content extracted from three marine microalgae species and the ORAC values tested. As seen in Table 1, in this work, no relationship was found between phenolic compound content and antioxidant activity (ROS-RNS); in fact, the less polar solvent did not always yield the best antioxidant activities, neither in ROS nor in RNS. 

Therefore, for understanding and a more precise evaluation of the antioxidant potential, it is crucial to consider more detailed information regarding the chemical composition, specifically analyzing carotenoid, phenolic, and lipid compounds.

### 2.2. PLE Optimization 

To obtain microalgae extracts with neuroprotective potential, two different PLE procedures were optimized according to the central composite design described above, taking into account different response variables and trends. Optimization considered the maximum response values, except for the IC50, where minimum values were desired. The first optimum was obtained considering AChE and RNS as response variables and provided the following conditions: 40 °C and 34.9% ethyl acetate (in CPME) (Optimum 1); the second optimum was selected for LOX, ROS, and yield, with the following condition: 180 °C and 54.1% ethyl acetate (in CPME) for (Optimum 2). Estimated response values are listed in Table 2. The levels of desirability obtained by maximizing all responses were 0.962 and 0.899 for the first and second optimization conditions, respectively. The optimal extraction conditions were experimentally tested in triplicate to validate the model prediction in terms of the evaluated responses. The experimental results obtained were compared with the values predicted by the quadratic model (Table 2). As can be seen, experimental data were in agreement with those predicted by the multiple response optimization for AChE-RNS (Optimum 1), and ROS-LOX-Yield (Optimum 2). Considering the interest of other cholinergic inhibitors (ChEIs) that can increase the activity of surviving cholinergic neurons in patients with AD, dual effects (AChE and BuChE) was investigated in our extracts obtained at optimum conditions. BuChE is known to play an established role in the regulation of acetylcholine (like AChE), but its action is more related to ‘nervous system–immune system interactions’ and inflammatory pathways [43].

As can be seen, the low-temperature extracts had similar inhibition of AChE and BuChE, whereas the high-temperature extracts showed an almost twofold difference in inhibition of AChE and BuChE. Although AChE and BuChE share approximately 55% of the amino acid sequence, they exhibit structural similarities and differences. This may cause different catalytic properties and may affect the inhibition status [43]. In conclusion, the IC50 values for AChE and BuChE (44.26 and 48.79 µg mL^−1^, respectively) of the extracts obtained from the extraction at 40 °C with 34.9% ethyl acetate (in CPME) indicate their high neuroprotective potential [29,30,44,45]. On the other hand, although the IC50 values (82.91 and 65.67 µg mL^−1^, respectively) for AChE and BuChE of the extracts obtained from the extraction with 180 °C and 54.1% ethyl acetate (in CPME) were higher, these results demonstrated better AChE and BuChE potential than many other studies [27,43,44,46,47].

The anti-inflammatory capacity of optimum extracts was investigated, and their LOX (lipoxygenase) inhibition IC50 values were determined as 22.56 µg mL^−1^ for optimum extract 1 and 11.59 µg mL^−1^ for optimum extract 2. In other words, extracts with higher anti-inflammatory capacity were obtained at high temperatures. A study evaluating the anti-inflammatory capacity of extracts from various microalgae through LOX inhibition revealed IC50 LOX values of 51.82 µg mL^−1^ for *H. pluvialis*, 28.45 µg mL^−1^ for *T. lutea*, 73.30 µg mL^−1^ for *P. cruentum*, and 50.05 µg mL^−1^ for *N. oceanica* [27]. Based on these results, it can be concluded that *T. chuii* is a promising source of anti-inflammatory compounds, particularly under the optimal extraction conditions involving higher temperatures. The results indicate that the optimum extracts obtained from *T. chuii* possess potent anti-inflammatory properties.

The antioxidant capacities of the optimized extracts were evaluated by performing ROS-ORAC and RNS assays targeting reactive oxygen species and reactive nitrogen species, respectively. The ORAC IC50 values for the first optimized extract and the second optimized extract were determined to be 9.74 µg mL^−1^ and 3.34 µg mL^−1^, respectively. In addition, the RNS IC50 values for the first optimized extract and the second optimized extract were determined to be 89.22 µg mL^−1^ and 120.46 µg mL^−1^, respectively.

These results clearly demonstrated that the first optimized extract, which was obtained under lower temperature conditions, exhibited remarkable RNS activity, as expected. Conversely, the second optimized extract obtained at higher temperatures showed superior ORAC activity. It is worth noting that RNS assays focus primarily on nitrogen species and use specific reagents or probes, while ORAC assays use fluorescent or chemiluminescent dyes and target oxygen-centered radicals. The evaluation of RNS assays is relevant to the study of biological processes associated with reactive nitrogen species, whereas ORAC assays provide a more comprehensive assessment of overall antioxidant capacity, which means that both assays measure the activity of different compounds. Therefore, it is crucial to use a combination of different assays to gain a comprehensive understanding of the antioxidant properties under investigation. Accordingly, the optimization process was carried out by considering different response variables, allowing the extraction of anticholinergic extracts (characterized by lower efficiency, and therefore not considered acceptable as the primary response variable), as well as antioxidant and anti-inflammatory extracts (characterized by higher efficiency). Given the complex nature of Alzheimer’s disease, it is imperative to discover compounds with diverse bioactivities. By targeting multiple pathological processes involved in the disease, we can potentially achieve more comprehensive therapeutic benefits. For example, compounds with both AchE and BuchE activity may help reduce the deposition of beta-amyloid plaques and inhibit the formation of neurofibrillary tangles, thereby addressing the underlying protein abnormalities in Alzheimer’s disease [4,5]. In addition, compounds with antioxidant properties can attenuate oxidative stress, while anti-inflammatory compounds can counteract the inflammatory processes associated with the disease. These dysfunctions are interrelated and may be mutually reinforcing. Targeting all three at the same time can result in synergistic effects, where the combined treatment has a greater impact than each treatment alone.

The concept of using a combined extract, such as Optimum 1 and 2, which outperforms the extract obtained under intermediate conditions, holds significant potential for improved efficacy in the treatment/prevention of Alzheimer’s disease. This strategy could be in line with personalized treatment trends, providing each individual with the right balance of extracts to re-balance the dysfunctions at each moment caused by the neurodegenerative disorder. The synergistic effects achieved by simultaneously targeting multiple factors influencing AD, including oxidation, inflammation, and cholinergic dysfunction, offer a more comprehensive and promising therapeutic strategy. 

In summary, the complex nature of Alzheimer’s disease requires a multifaceted approach to therapeutic intervention. By targeting multiple pathological processes and incorporating diverse bioactive compounds, we can potentially provide more comprehensive treatment benefits. Further research and development in this area is essential to advance our understanding and improve the efficacy of therapeutic strategies for Alzheimer’s disease.

**Figure 2:**
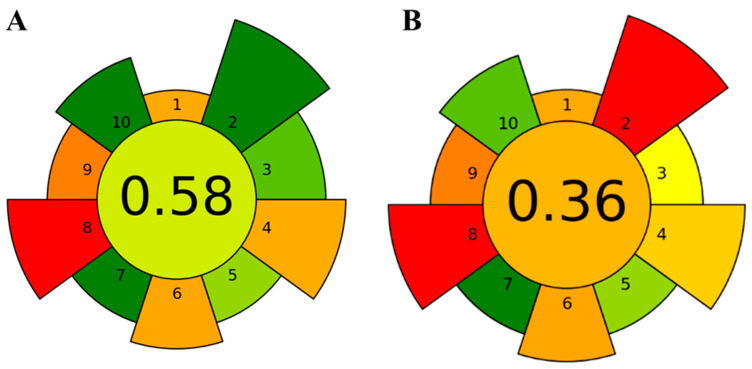
The results of the Analytical Greenness Metric for Sample Preparation (AGREEprep 0.91) assessment of the procedure of optimized PLE for neuroprotective extracts production using green solvents (**A**) and hexane (**B**). The 10 criterion description can be seen in [48]; default weights were used.

A green metric evaluation of the extraction process was performed using the Analytical Greenness Metric for Sample Preparation (AGREEprep). AGREEprep [48] is a freely available and user-friendly software that generates a visually understandable representation illustrating the overall performance and structure of the developed methodology, software can be downloaded from http://mostwiedzy.pl/AGREEprep (version 0.91 was used in this paper, last accessed 1 February 2024). The AGREE diagram obtained is shown in Figure 2. The center of the diagram shows the greener score of the process, with 1 being totally green. In our case, 0.58 was the score using green solvents (A), while the same procedure using hexane had a score of 0.36 (B).

The on-site extraction process used a solvent mixture of 25 mL. However, it was assumed that the solvents used were sustainable or renewable. In addition, after evaporation, the solvents could be recovered for future use, although only 75% of the volume was considered suitable for reuse, resulting in 6.25 mL being classified as waste or emissions. In accordance with the fifth principle of Green Analytical Chemistry, our procedure used a small sample size of 1 g, effectively reducing time, effort, cost, and resource consumption. In addition, the extraction procedure was automated, in accordance with criterion 8 of Green Analytical Chemistry. However, due to the limitations of the instrumentation used, only three extractions per hour were possible, although certain brands allow up to six simultaneous extractions, so this point can even be improved. The ASE200 instrument (Thermo Fisher Scientific, Sunnyvale, CA, USA) used in our study consumed approximately 800 W per extraction due to heating and pumping. The high-performance liquid chromatography with diode array detection and mass spectrometry (HPLC-MS/MS) technique was then used in the next procedural step. The overall AGREEprep score obtained was 0.58, indicating a moderate level of analytical greenness for the proposed method. This green metric tool did not find differences in the two optimal conditions studied, since both used green solvents and similar energy consumption.

### 2.3. Supercritical Fluid Extraction and Combined SFE-PLE Biorefinery Process 

**Figure 3:**
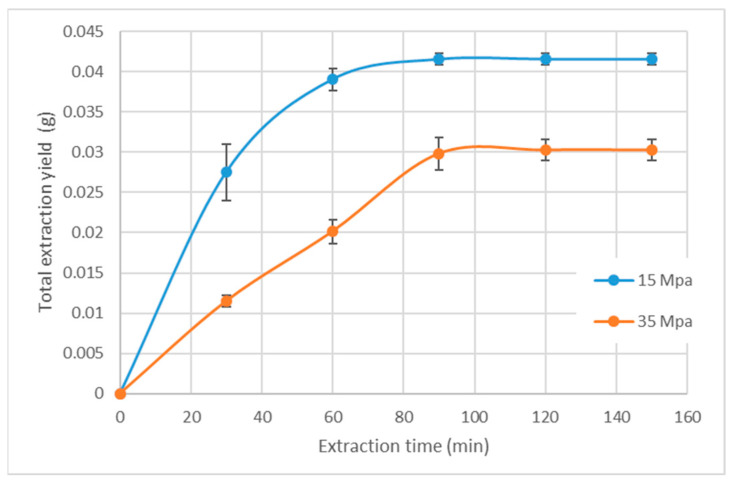
Kinetic study of supercritical fluid extraction applied to *T. chuii* biomass.

Once the optimized conditions for PLE were determined, the effect of a non-polar green solvent, such as carbon dioxide (CO_2_), was studied. In this regard, the biological activity of the extracts obtained by supercritical fluid extraction (SFE) was studied under two different densities. These density variations were obtained by maintaining the same extraction temperature (40 °C) but changing the pressures to 15 MPa (780.3 kg/m^3^) and 35 MPa (934.9 kg/m^3^). To ensure a consistent extraction time, a kinetic study was performed. This study determined the optimal extraction time by focusing on the initial phase of the extraction curve where the total yield reached an asymptotic state. This optimal extraction time was found to be 90 min, as shown in Figure 3. Moreover, in this figure, it can be clearly seen that extraction yield was higher using low CO_2_ density, which can be attributed to raw material compactation at higher pressure. This effect has been previously depicted in other raw materials such as spearmint [49] or mango [50].

Once the extraction time was determined, a study of the bioactivity of the extracts was conducted. The results, as shown in Table 3, clearly demonstrated that the anti-inflammatory activity, as assessed by LOX inhibition, as well as the anticholinesterase activity, showed remarkable similarity under both extraction conditions. However, it is noteworthy that the antioxidant activity, as indicated by the lower IC50 value, was significantly higher when the extract was obtained using a lower density.

Unfortunately, the sequential process based on compressed fluids for the biorefinery of bioactive compounds present in *T. chuii* did not provide the desired results. Despite the application of both the optimized extraction methods shown in Table 2 to the unextracted material obtained from the low-density supercritical fluid extraction (150 bar 40 °C), the bioactivities discovered were inferior to those observed using individual extraction processes. Unfortunately, neither the IC50 value for ACHE nor for ORAC could be determined. Moreover, the extracts obtained from the sequential process showed approximately 60 times less anti-inflammatory activity compared to the pure PLE extracts. Therefore, it can be concluded that the combination of SFE + PLE did not increase the bioactivity of the extracts. 

### 2.4. Chemical Characterization

#### 2.4.1. GC-QTOF-MS Profiling Analysis 

A comprehensive lipidic profiling analysis of the two optimal PLE extracts was performed by GC-QTOF-MS. PLE extracts were derivatized to improve the detectability of fatty acids and other lipidic metabolites such as phytosterols, tocopherol, and other terpenoids. Using the derivatization procedure described above, trimethylsilyl (TMS) and tert- butyl dimethyl (TBDMS) derivatives were identified applying an automated full-scan MS data-mining process, which integrates peak detection, deconvolution, and MS database search. Following this approach, a total of 34 lipidic compounds, including saturated fatty acids (FA), monounsaturated fatty acids (MUFAs), and polyunsaturated fatty acids (PUFAs), as well as relevant terpenoids and sterols (Table 4), were tentatively identified based on the positive match of the experimental mass spectra with MS databases (i.e., NIST and Fiehn lib); exact mass values, as determined by HRMS; and data reported in the literature. GC-QTOF-MS parameters such as retention time, generated molecular formula, match factor values from the MS database search, and main HRMS fragments were considered for annotation. Identification reliability was considered satisfactory for chemical structures showing match factor values above 70. 

The amount of MUFAs and PUFAs in the analyzed extracts accounted for 74–78% of the total fatty acid content (TFA) in equal proportion (Supplementary Table S1). The content of MUFAs (expressed as % of TFA) was mainly represented by 24% oleic acid (C18:1n-9), 4% palmitoleic acid (C16:1n-7), and 4% (Z)-11-eicosenoic acid (C20:1n-9). Major PUFAS were 15–17% α-linolenic (C18:3n-3; ALA) and 16% eicosapentaenoic acids (C20:5n-3; EPA), followed by 3–4% linoleic acid (C18:2n-6; LA) and 1% arachidonic acid (C20:4n-6; ARA). The saturated fatty acid profile was mainly composed of 18–22% palmitic acid (C16:0;), followed by other minor long-chain saturated FA such as capric acid (C10:0), lauric acid (C12:0), myristic acid (C14:0), pentadecanoic acid (C15:0), stearic acid (C18:0), and lignoceric acid (C24:0).

Microalgae like *T. chuii* are a natural source of PUFAs with health-promoting properties in different neurodegenerative pathologies, including Alzheimer’s disease (AD) [40,41]. Studies with different strains of *T. chuii* have reported a high content of PUFAs, particularly EPA, ALA, ARA, and docosahexaenoic acid (DHA), although the latter was detected in certain strains and growing phases [14,51,52]. The optimal PLE extracts in this work contained interesting molecules for nutraceutical uses, such as omega-3 (e.g., EPA and ALA) and omega-6 fatty acids (e.g., LA and ARA). In particular, omega-3 fatty acids can play a vital role in Alzheimer’s disease progression due to the neuroprotective potential associated to the anti-inflammatory effect [53]. In this line, PUFAs from *Nannochloropsis oceanica* have been reported as protective agents against cognitive impairment induced by amyloid-β, and against oxidative stress in the brain [45]. Therefore, the presence of high levels of PUFAs in the obtained PLE extracts may contribute to its neuroprotection capacity. The omega-3/omega-6 ratio of these extracts is over 1:1, ranging from 6.9 to 7.4, which validates the nutritional value of strains for food supplements [54]. Considering all omega-3 PUFAs, our PLE extracts presented values (32–34% of TFA) in the average range of those observed for other *Tetraselmis* species (18–68%) and for other microalgae (12–64%) extracts. Conversely, our MUFA content (39%) was above the range observed for other *T. chuii* extracts (14–30%) [52].

The optimal PLE extracts were also shown to be a source of bioactive terpenoids, including phytosterols (e.g., campesterol and isofucosterol), neophytadiene, and α-tocopherols (vitamin E). Some studies suggest that vitamin E supplementation may have beneficial effects on cognitive function and Alzheimer’s disease. For example, vitamin E was shown not only to enhance cognitive function but also to reduce brain inflammation in Alzheimer’s disease models [55]. Further studies with AD animal models demonstrated that combined administration of vitamin E and fish oil at low dosages improved cognitive function [56]. On the other hand, phytosterols were demonstrated to act as AChE inhibitors, exhibiting the capacity to cross the blood–brain barrier (BBB) [34,57]. Therefore, phytosterols can play an important role in neurodegenerative diseases research, and their presence in the target PLE extracts might help to explain their neuroprotective potential.

#### 2.4.2. UHPLC-DAD-QTOF-MS/MS Profiling Analysis 

The optimal PLE extracts from *T. chuii* were analyzed by ultra-high-performance liquid chromatography (UHPLC) using a diode array detector (DAD) coupled in line with quadrupole time-of-flight (QTOF) and mass spectrometry (QTOF-MS) detection to determine the profile of carotenoids, chlorophylls, and other lipids. To cover a wide range of chemical structures and obtain complementary structural information, ultra-violet visible (UV-VIS) profiles and QTOF-MS/MS data, acquired in positive and negative ionization mode, using both electrospray ionization (ESI) and atmospheric pressure chemical ionization (APCI) sources, were jointly analyzed.

**Figure 4:**
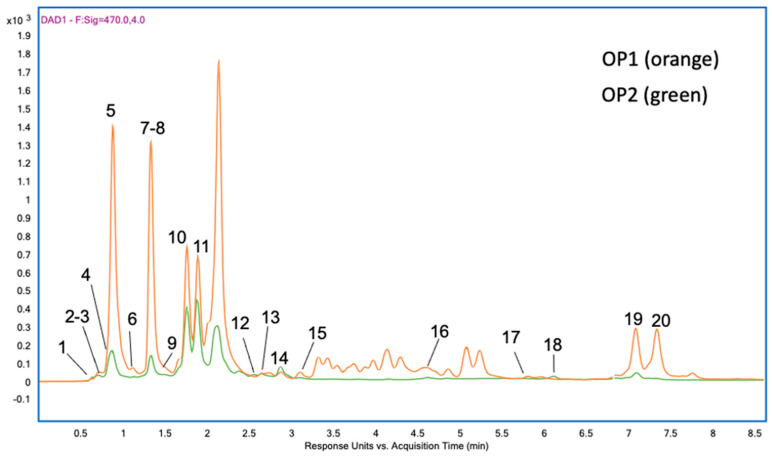
LC-DAD analysis (470 nm) of *Tetraselmis chuii* extracts (OP1 and OP2; extraction conditions shown in Table 2) obtained under optimal PLE conditions. The names of compounds from **1** to **20** can be found in Table 4.

The UHPLC-DAD profile at 470nm (Figure 4) revealed the abundant presence of pigments related to photosynthesis, such as carotenoids and chlorophylls, in the optimized PLE extracts. Twenty major peaks were tentatively identified according to their maximum absorption wavelength (λmax), molecular ion (*m*/*z*), and main MS/MS fragments obtained by LC-APCI(+)-MS/MS analysis. Structural information of the annotated pigments is summarized in Table 5. From their calculated molecular formulae, compounds **1** to **11** belong to the xanthophyll class (oxygen-containing carotenoids), whereas compounds **19** and **20** were classified as carotenes (hydrocarbon carotenoids). The nitrogen-containing pigments (compounds **12** to **18**) were classified as chlorophylls and chlorophyll derivatives.

Carotenoids 1 to 9, 19, and 20 showed three typical maximum absorption wavelengths ranging from 400 to 475 nm in their UV-VIS spectra. Compounds **3** and **5** are isomers with molecular ion at *m*/*z* 601.4251 (C40H56O4), which were annotated as neoxanthin and violaxanthin, respectively. The two epoxycarotenoids contain three and two hydroxy groups, respectively, which justifies their relative retention time. Three additional epoxycarotenoids (compounds **1**, **4,** and **9**) were annotated as fucoxanthinol (*m*/*z* 617.4201, C40H56O5), diadinoxanthin (*m*/*z* 583.4146l, C40H54O3), and antheraxanthin (*m*/*z* 585.4432, C40H56O3). The lower retention time of fucoxanthinol is consistent with its higher oxygenation degree. Among the identified hydroxycarotenoids, compounds **2**, **6**, **7,** and **8** were annotated as prasinoxanthin (*m*/*z* 601.4251, C40H56O4), diatoxanthin (*m*/*z* 582.4073, C40H54O3), zeaxanthin/lutein (*m*/*z* 569.4353, C40H56O2), and crocoxanthin (*m*/*z* 551,4213, C40H54O), respectively. Additionally, two non-oxygenated carotenoid isomers (compounds **19** and **20**) were identified as α- and β-carotene (*m*/*z* 537.4455, C40H56) at higher retention times, in agreement with their higher lipophilicity. Unlike the abovementioned carotenoids, compounds **10** and **11** showed a single λmax value at 455–460 nm in their UV-VIS spectrum, characteristic of ketocarotenoids like echinenone (*m*/*z* 567.4197, C40H54O2). 

Chlorophylls and their derivatives exhibit two major absorption bands in the visible range, corresponding to the cyclic tetrapyrrole (porphyrin) skeleton, at around 420–460 and above 650 nm. Due to operational restrictions, only the first band could be measured in this work. In agreement with λmax values in the literature, compounds **14** and **15** were annotated as chlorophyll a and chlorophyll b, corresponding to molecular ions at *m*/*z* 893.5426 (C55H72MgN4O5) and *m*/*z* 907.5218 (C55H70MgN4O6), respectively. Two chlorophyll a derivatives were also identified as divinylchlorophyll a (compound **12**) and 7-hydroxychlorophyll a (compound **13**) at *m*/*z* 891.5270 (C55H70MgN405) and *m*/*z* 891.5270 (C55H72MgN4O6), respectively. Two additional derivatives lacking the central Mg-atom were annotated as pyropheophytin b (compound **16**) and pheophytin a (compound **17**). These demetalated forms are less polar than the corresponding chlorophylls, showing higher retention time values in reverse phase columns. The most abundant fragment ion MS/MS spectra of chlorophyll and its derivatives usually correspond to the fragmentation with the loss of the phytil chain [M-278]+.

The comparative profiles of pigments identified in the two optimal PLE extracts from *T. chuii* revealed a higher content of carotenoids and chlorophylls operating at 40 °C (OP1), compared to extracts obtained at 180 °C. This can be explained by considering that the stability of carotenoids is expected to decrease with temperature [57]. The main carotenoids in the target extracts were neoxanthin, violaxanthin, and zeaxanthin/lutein, followed by echinenone and α- and β-carotene. The profile of identified carotenoids in these extracts is in line with data reported in literature for *T. chuii* extracts obtained at the lab scale [14,51,58,59]. In the analysis of supercritical fluid and combined supercritical fluid (SFE) and pressurized liquid extraction (SFE + PLE) extracts, a comparison was made with the previously optimized pressurized liquid extraction (PLE) extract. It was observed that the SFE extract lacked chlorophylls, whereas the extract obtained by the combined SFE + PLE process lacked nonpolar carotenoids. Both the SFE and the combined SFE + PLE extracts showed lower bioactivities compared to the optimal PLE extracts. Therefore, it can be concluded that the observed activity may have been due to synergistic effects between carotenoids and chlorophylls.

Carotenoid-rich extracts have been shown to inhibit Aβ42 aggregation. For instance, Gallego et al. [60] reported antioxidant, anti-inflammatory, and anticholinergic activities of a carotenoid-enriched extract from *Dunaliella salina* microalgae, as well as a neuroprotective effect against neurotoxic agents Aβ1-42 and L-glutamate in a neuron-like cell model. Our research group also demonstrated the anti-cholinesterase, antioxidant, and anti-inflammatory potential of extracts enriched in carotenoids from various microalgae. In addition, they determined that while all microalgae extracts showed an anti-inflammatory effect by attenuating LPS-induced inflammation in THP-1 cells, some extracts significantly reduced the release of all identified cytokines [27].

The capacity of carotenoids to cross the blood–brain barrier (BBB) is, to some extent, contingent upon factors such as the specific carotenoids, their concentration, and the overall health and integrity of the BBB, thereby exhibiting variability [61,62]. Given the significance of carotenoids in neuronal protection against oxidative stress and inflammation, which are implicated in the development and progression of Alzheimer’s disease, it is imperative to conduct comprehensive research to fully understand their mechanism of action and therapeutic potential in this specific context.

The connection between lipids and Alzheimer’s disease is intricate and not entirely understood. Therefore, a comprehensive lipidomic profile of the obtained PLE extract also was carried out to gain further insight into the potential impact of *T. chuii* on neurogeneration. The results of the UHPLC-ESI-Q-TOF-MS analysis revealed the presence of a total of 60 major lipids, including glycerolipids, phospholipids, sphingolipids, and isoprenoids (Table S1). 

**Figure 5:**
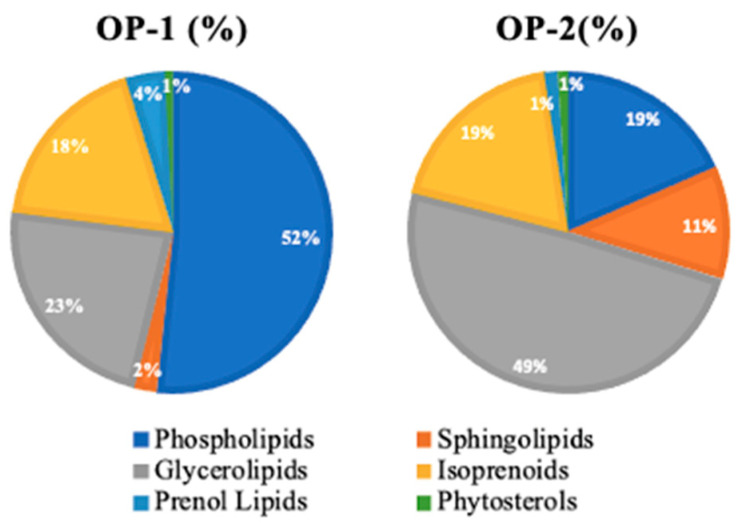
The concentrations of lipids in optimum *Tetraselmis chuii* extracts (OP1 and OP2; extraction conditions are shown in Table 2) were determined using UHPLC-ESI(+)-QTOF-MS analysis.

Figure 5 illustrates the comparative analysis of the examined extracts, revealing similar levels of phytosterols and isoprenoids (18.285% and 0.701% for OP1, 18.775% and 0.973% for OP2, respectively). Additionally, OP-1 extract exhibited high concentrations of phospholipids (51.599%) and prenol lipids (4.028%), while OP-2 extract demonstrated richness in glycerolipids (49.205%) and sphingolipids (11.328%).

These lipids have also been studied for their potential effects on Alzheimer’s disease, as important components of cell membranes, playing crucial roles in maintaining the structure and function of brain cells. Studies have shown that changes in the composition of cell membranes, including alterations in the levels of phospholipids, glycolipids, and cholesterol, can occur in Alzheimer’s disease [63].

These changes can affect the function of important proteins, such as those involved in neurotransmitter release and synaptic plasticity, which can contribute to cognitive decline [64]. For instance, the levels of certain sphingolipids were shown to decrease in the brains during Alzheimer’s disease, and these changes are associated with neuronal dysfunction and cognitive impairment [65]. In this regard, the effectiveness of a micronutrient supplementation containing carotenoids, omega-3 fatty acids, and vitamin E on Alzheimer’s disease was measured in a 12 month study. The active group (*n* = 50) who received the micronutrient supplementation performed significantly better than the placebo group (*n* = 27), with memory showing the most improvement [66]. These pieces of evidence support that *T. chuii* extracts obtained in this work represent a food source rich in bioactive non-polar compounds such as carotenoids, terpenoids, fatty acids, and sterols, with strong potential as neuroprotective agents against Alzheimer’s disease. 

### 2.5. Cytotoxicity Evaluation of T. chuii Extracts

An additional step in advancing the use of extracts with neuroprotective potential is to conduct in vitro toxicity evaluations to assess their cytotoxic effects using cell culture models. These models provide valuable opportunities to investigate the potential bioactive properties of natural extracts and can help predict their behavior in more complex organisms, including animals and humans. In this particular study, the researchers aimed to investigate the in vitro cytotoxicity of the optimal PLE *T. chuii* extracts using the human HK-2 cell line and a differentiated form of THP-1 cells, which is a widely used human monocytic cell line commonly used to study immune system disorders and inflammation [67,68]. HK-2 cells refer to a specific cell line called Human Kidney-2. They are immortalized human proximal tubular epithelial cells that are widely used in the study of kidney biology and disease. HK-2 cells are derived from normal human kidney tissue and are widely used as a model system to study various aspects of renal physiology and renal toxicology, and they are a common model to study cytotoxicity. THP-1 cells are a well-known cell line derived from a human monocytic leukemia patient. THP-1 cells exhibit characteristics of monocytes and macrophages, making them an excellent model for studying immune system disorders and inflammatory responses, as well as evaluating potential therapeutic interventions. THP-1 cells are widely used in both basic and translational research. They can be induced to differentiate into macrophage-like cells. This differentiation process leads to changes in cellular morphology and the acquisition of macrophage-like functions, allowing studies of cellular responses, phagocytosis, cytokine production, and other immune-related processes. In addition, these cells serve as an accessible platform to evaluate the efficacy of anti-inflammatory agents, immunomodulatory compounds, and potential immunotherapies. 

**Figure 6:**
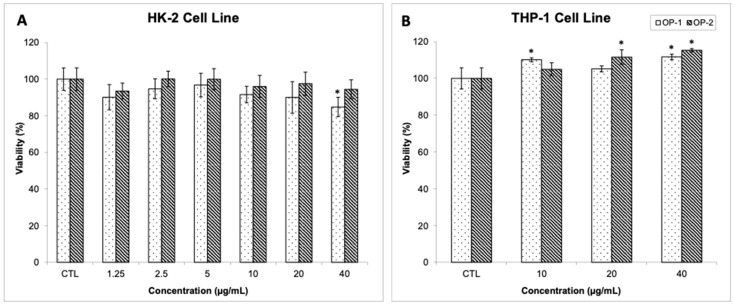
Cell viability (%) of human kidney HK-2 cells (**A**) and human monocytic leukemia THP-1 cells (**B**) after exposure to different concentrations of two PLE *Tetraselmis chuii* optimum extracts (OP1 and OP2; extraction conditions are shown in Table 2) for 24 h. Error bars are given as the standard deviation of three independent experiments; asterisks indicate significant differences by *t*-test (*p*-value < 0.05).

The data obtained, as shown in Figure 6A,B, revealed that both extracts exhibited negligible cytotoxic effects up to a concentration of 40 μg mL^−1^, with the exception of OP-1 extract, which showed a slight cytotoxic effect on HK-2 cells. Furthermore, it was interesting to observe that both extracts showed the ability to promote the proliferation of THP-1 cells at the highest concentration tested. Taken together, these results suggest that both PLE *T. chuii* extracts are safe for further investigation. However, to specifically evaluate their neuroprotective capacity, future in vitro studies will be conducted using other cell culture models such as neuroblastoma. Expanding the scope of analysis to include additional cell culture models will provide further insight into the potential benefits and applications of these extracts. Overall, this study represents an important advancement in the field of neuroprotection and lays the foundation for further exploration of the bioactive properties of PLE *T. chuii* extracts. By employing rigorous in vitro toxicity evaluations and expanding the investigation to a variety of cell culture models, researchers are paving the way to unlock the full potential of these extracts for neuroprotective applications.

## 3. Materials and Methods

### 3.1. Materials

*Tetraselmis chuii* was kindly donated by Fitoplancton Marino S.L. (El Puerto de Santa María, Cádiz Spain). *T. chuii* dried powder was vacuum-packed (C400 Multivac. Wolfertschwenden, Germany) and stored at −18 °C. HPLC-grade solvents ethyl acetate (ETAC), ethanol (EtOH), and cyclopentyl methyl ether (CPME) were purchased from VWR Chemicals (Barcelona, Spain). Acetylcholinesterase (AChE) Type VI-S from Electrophorus electricus, butyrylcholinesterase from equine serum (BuChE), acetylthiocholine iodide (ATCI), linoleic acid (LA), 2,2′-azino-bis (3-ethylbenzothiazoline-6-sulphonic acid) (ABTS), Trizma hydrochloride (Tris-HCl), disodium phosphate (Na_2_HPO_4_), monopotassium phosphate (KH_2_PO_4_), sodium nitroprusside dehydrate (SNP), fluorescein sodium salt, sulphanilamide, naphthylethylene diamine dihydrochloride, gallic acid, ascorbic acid, quercetin, lipoxidase from Glycine max (soybean), 4-(amino- 359 sulfonyl)-7-fluoro-2,1,3-benzoxadiazole (ABD-F), galantamine hydrobromide, and 2,2-azobis(2-amidinopropane) dihydrochloride (AAPH) were purchased from TCI Chemicals (Tokyo, Japan). Ultrapure water was obtained from a Millipore system (Billerica, MA, USA). 

Human proximal tubular epithelial cells (HK-2) and human THP-1 monocytes were from ATCC (Rockville, MD, USA). Dulbecco’s modified Eagle’s medium nutrient mixture (DMEM/F12) and Roswell Park Memorial Institute (RPMI 1640) culture media, fetal bovine serum (FBS), PBS, L-glutamine, antibiotic solution (including penicillin and streptomycin), antibiotic–antimycotic solution (including penicillin, streptomycin, and amphotericin B), and insulin–transferrin–selenium solution were purchased from Thermo Fisher (Grand Island, NY, USA). 3-(4,5-Dimethylthiazol-2-yl)-2,5-diphenyltetrazolium bromide (MTT), phorbol 12-myristate 13-acetate (PMA), and β-mercaptoethanol were obtained from Sigma-Aldrich (St. Louis, MO, USA).

### 3.2. Method

#### 3.2.1. Optimization of the Pressurized Liquid Extraction (PLE) Procedure 

In the present study, ETAC and CPME were chosen as PLE solvents, and the effect of the temperature and solvent composition was evaluated on the extraction yield with a set of in vitro bioactivities involved in the neuroprotective potential. Two factors were considered at three levels: temperature (40, 110 and 180 °C) and solvent (CPME (100%), ETAC (100%), and CPME:ETAC (50:50, *v*/*v*)). With only one extraction cycle, the pressure and total extraction time were fixed at 10.5 MPa and 20 min, respectively. The extraction was carried out by loading 1.0 g of dried algae biomass into an 11 mL stainless steel extraction cell, sandwiched between two layers of sea sand (each 2.0 g), which are chemically inert, providing balanced pressure distribution and physical support to enhance extraction efficiency due to their porous structure. Extraction was carried out using a Dionex accelerated solvent extractor (ASE 200, Sunnyvale, CA, USA). One minute of nitrogen purging at 9 bar was applied to push any residual solvent from exhausted algae powder residue. Finally, the extracts obtained were collected in 40 mL glass vials, dried using a gentle stream of nitrogen, protected from light, and stored at −20 °C until further analysis.

To optimize the extraction of neuroprotective compounds from the algae mass, a response surface methodology (RSM) was proposed, using a central composite design (CCD). Response variables studied were extraction yield, enzymatic inhibition activity (AChE, BuChE, and LOX), and antioxidant capacity (ROS and RNS), as described below. The extraction yield was expressed as the percentage of extract dry weight per initial powder algae mass dry weight. After the extraction process with the solvents, the liquid extracts were dried under nitrogen flow and, after they were completely dry, they were weighed. Therefore, the equation was determined by a relationship between the extracted mass (Em) and algae mass used (Am), as observed in the following Equation (1).

Yield (%) = Em/Am × 100(1)

The quadratic equation model for each variable (Yi) was (Equation (2)):

Yi = β_0_ + β_1_t + β_2_T + β_1,2_ T × t + β_1,1_ t^2^ + β_2,2_ T^2^ + error (2)

where t is the extraction time; T is the temperature; β_0_ is the intercept; β_1_ and β_2_ are the linear coefficients; β_1,2_ is the linear-by-linear interaction coefficients; and β_1,1_ and β_2,2_ are the quadratic coefficients and the error variable, respectively. Data analysis for experimental design and multi response optimization was performed using Statgraphics Centurion XVII software (StatPoint Technologies, Inc., Warrenton, VA, USA). The analysis of variance (ANOVA), standardized Pareto charts, coefficient of determination (R2), response surfaces, *p* values for the model, interaction plot, and lack-of-fit testing were analyzed, accepting significances at *p* ≤ 0.05.

#### 3.2.2. Optimization of the Supercritical Fluid Extraction (SFE) Procedure

In order to study the bioactivity of the less polar fraction, carbon dioxide was used as the extraction solvent under supercritical conditions. Extractions were carried out using the same home-made equipment as described in Fagundes et al. [34] that consisted of a homemade compressed fluid extractor coupled to a high-pressure pump (PU-2080 Plus CO_2_; Jasco, Hachioji, Japan) and solvent pump (PU-2080; Jasco Plus, Hachioji, Japan). Extractions were performed at 40 °C, 4 mL CO_2_/min (measured in the pump head at −3 °C and 5 MPa), using 90 min of extraction time and 1 g of dried microalga mixed with 4 g of sea sand. Two pressures were used (15 and 35 MPa) in order to obtain different CO_2_ densities.

#### 3.2.3. Chemical Characterization

##### Gas Chromatography–Mass Spectrometry Analysis (GC-QTOF-MS)

The analysis of the extracts was performed employing an Agilent 7890B gas chromatography (GC) system coupled to an Agilent 7200 quadrupole time-of-flight (Q-TOF) mass spectrometer, equipped with an electronic ionization (EI) interface. The separation was carried out using an Agilent Zorbax DB5-MS Column (30 m × 250 μm i. D. × 0.25 μm) + 10 m DuraGuard capillary column. Helium was used as the carrier gas at a constant flow rate of 0.8 mL min^−1^. The injection volume was 1 μL. Splitless mode was used for injection, keeping the injector temperature at 250 °C. The GC oven was programmed at 60 °C for 1 min, then increased at a rate of 10 °C min^−1^ to 325 °C, and was held at this temperature for 10 min. MS detector was operated in full-scan acquisition mode at a *m*/*z* scan range of 50–600 Da (5 spectra per second). The temperatures of the transfer line, the quadrupole, and the ion source were set at 290, 150, and 250 °C, respectively. 

To identify the lipids present in the samples, the extracts underwent derivatization using the method outlined by Fiehn [69]. A 40 mg mL^−1^ solution of MeoX in pyridine was prepared. The dried samples were dissolved by adding 10 μL of the MeOX solution, vortexed, and transferred to the Thermomixer. The samples were derivatized by adding a mixture of MSTFA (N-methyl-N-(trimethylsilyl)trifluoroacetamide) and d27-n-myristic acid, and agitation was performed in the Thermomixer. The contents were transferred to sealed glass vials with inserts. A waiting period of 2 h was observed before the samples were injected into the GC-MS.

Target compounds were annotated by systematic mass spectra deconvolution and searched in the MS database, using the Agilent MassHunter Unknowns Analysis tool (version 12.0) and NIST MS database search.

##### Liquid Chromatography–Mass Spectrometry Analysis (HPLC-DAD-MS/MS) 

Carotenoid and pigment identification was performed by HPLC-DAD-APCI-QTOF-MS/MS. The analysis of the extracts was carried out in an Agilent 1290 UHPLC system equipped with a DAD, coupled to an Agilent 6540 q-TOF/MS equipped with an APCI source, all from Agilent Technologies (Santa Clara, CA, USA). A Thermo Fisher Scientific Accucore C30 column (2.6 μm, 4.6 × 50 mm) was used at 30 °C. Separation was achieved using a gradient program starting with 100% mobile phase A (90% Methanol, 7% MTBE, 3% water) and 0% mobile phase B (90% MTBE, 10% methanol) changing to 100% mobile phase B within 12 min. This was kept constant for 1.5 min before returning to the initial conditions within 1.5 min. The total run time was 15 min at a flow rate of 0.8 mL/min. The mass spectrometer was operated in positive ionization mode (APCI+), with gas temperature of 300 °C; drying gas of 8 L/min; vaporizer temperature of 350 °C; nebulizer pressure of 40 Psi; capillary voltage of 3500 V; corona+: 4 μA; fragmentor voltage of 110 V; and skimmer voltage of 45 V. The MS and auto MS/MS modes were set to acquire *m*/*z* values ranging between 25 and 1500, at a scan rate of 10 spectra per second. Auto MS/MS mode was operated at two collision-induced dissociation energies: 20 and 40 eV, and selecting 4 precursor ions per cycle at a threshold of 200 counts. 

The lipidomic profile was carried out by HPLC-ESI-QTOF-MS/MS. The abovementioned LC-QTOF-MS system from Agilent, equipped with an orthogonal ESI source (Agilent Jet Stream, AJS) operating in positive ionization mode, was used. Chromatographic separation was carried out using a Zorbax Eclipse Plus C18 column (2.1 × 100 mm, 1.8 µm particle diameter), operating at a flow rate of 0.5 mL/min, 30 °C, and 20 μL injection volume. The mobile phases were composed of water (0.1% formic acid, solvent A) and acetonitrile (0.1% formic acid, solvent B). The elution gradient was as follows: 0% B at 0 min, 80% B at 12 min, 100% B at 14 min, 100% B at 16 min, and 0% B at 17 min. The mass spectrometer was operated in MS and MS/MS modes with a capillary voltage of 3000 V, nebulizer pressure of 40 psi, drying gas flow rate of 11 L/min, gas temperature of 300 °C, skimmer voltage of 45 V, and fragmentor voltage of 110 V. The MS and Auto MS/MS modes were configured to acquire *m*/*z* values ranging from 50 to 1100 and 50–800, respectively, at a scan rate of 5 spectra per second.

#### 3.2.4. In Vitro Bioactivity Assays

The natural extracts obtained from *T. chuii* biomass were studied by a battery of in vitro assays related to AD, such as reduction of cholinergic (AChE), anti-inflammatory (LOX), and antioxidant (ROS and RNS) activities. Those assays were previously tested in our lab with other microalgae [70,71,72].

##### Anti-Cholinergic Activity Assay

The inhibitory effect of microalgae extracts on AChE in vitro was determined according to the modified Ellman method [73] by a fluorescent enzyme kinetics study using a fluorescent probe, ABD-F, and galantamine was used as a positive control. A fluorescent method was chosen to avoid interferences in the absorbance reading associated with the presence of carotenoids and chlorophylls. The extract concentrations used for AChE testing range from 25 to 250 μg mL^−1^ in EtOH/H_2_O (1:1, *v*/*v*) with seven different concentrations. The fluorescence kinetic measurement was performed using a microplate reader (λexcitation and λemission were 389 nm and 513 nm, respectively), measuring at intervals of 1 min during 15 min, at 37 °C. The percentage of inhibition (inhibition degree, (ID)) was calculated with Equation (3).

(3) $$\%Inhibition=\frac{V0-V1}{V0}\times 100$$

*V*0 and *V*1 are the average velocity obtained for AChE in the absence and presence of the sample, respectively. The IC50 value represents the sample concentration (in μg mL^−1^) at which the cholinergic enzyme inhibitory capacity is inhibited by 50% compared to the control (without inhibitors), thus implying that lower IC50 concentrations show a greater inhibitory effect compared to higher IC50 values. Measurements were made in triplicate and results are expressed as mean ± standard deviation (SD).

##### Antioxidant and Scavenging Radical Capacity Assays: ROS and RNS

Reactive nitrogen species (RNS) scavenging capacity: RNS was evaluated by the nitric oxide (NO^•^) radical scavenging assay of microalgae extracts, according to Ho et al. [74], based on the reaction of Griess. The 96-well microplate filling distribution was as follows: 100 μL of extract sample at different concentrations (250–2500 μg mL^−1^) in EtOH/H_2_O (1:3, *v*/*v*), and 50 μL of SNP (5 mM) in buffer (30 mM pH = 7.5). The mixture was incubated at room temperature for 2 h under direct light. Nitrile ion concentration was measured at 734 nm after the addition of 100 μL ascorbic acid was used as the reference standard. Each measurement was carried out in triplicate, and the capacity of the extract for scavenging NO^•^ radicals was calculated through inhibition percentage as described in Equation (4).

(4) $$\%Inhibition=\frac{ARNSCONTROL-\left(ASAMPLE-ASAMPLE\;BLANK\right)}{ARNSCONTROL}$$

Reactive oxygen species (ROS) scavenging capacity—ORAC assay: The oxygen radical absorbance capacity (ORAC) method was performed according to Ou et al. [75]. In brief, analysis was performed by the reaction initiated by adding different concentrations (5–50 μg mL^−1^) in EtOH/H_2_O (1: 9, *v*/*v*), 100 μL of AAPH (590 mM) in PBS buffer (30 mM, pH = 7.5), 25 μL of fluorescein (10 μM) in PBS buffer, and 100 μL PBS buffer into a 96-well black polystyrene plate reader. Ascorbic acid was used as a reference.

Fluorescence kinetic measurements were recorded at λexcitation = 485 nm and λemission = 530 nm at 5 min intervals for a 60 min run time at 37 °C.

Each measurement was performed in triplicate, and the extract’s capacity to scavenge peroxyl radicals produced by spontaneous degradation of AAPH was calculated by means of the percent inhibition (AUCsample) difference between the area under the curve (AUC) of fluorescent degradation in the presence or absence of the sample with Equation (5).

(5) $$\%Inhibition=\frac{AUCcontrol-AUCsample}{AUCcontrol}\times 100$$

AUC was calculated by mean Equation (6):

(6) $$AUC=0.5+\sum \frac{fi}{f0}$$

where *f*0 and *fi* are fluorescence measurements at t = 0 min and every 5 min, respectively.

##### Anti-Inflammatory Activity Assay

Anti-inflammatory activity was measured by determining lipoxidase (LOX) inhibitory capacity using a fluorescent-based probe to observe enzyme kinetics, according to Whent et al. [76]. Analysis was performed after adding 100 µL of LA as substrate in EtOH/H_2_O (0.25:1, *v*/*v*), 100 µL of extract at seven different concentrations for each sample (21.5 to 215 μg mL^−1^) in EtOH/H_2_O (0.25:1, *v*/*v*), 75 µL of fluorescent (2 µM) in buffer, and 75 µL of LOX enzyme 0.0208 U/µL in buffer (Tris HCl, pH 9.0, 150 mM) into each well. Quercetin was used as a positive reference. Fluorescence kinetic measurement was performed on a microplate reader at λ_excitation_ = 485 nm and λ_emission_ = 530 nm, run time 15 min, 1 min, and 25 °C set temperature to reach the V_mean_ value of the enzymatic reaction. V1 and V0 are the mean velocity for LOX in the presence and absence of inhibitors, respectively. The equation represents the percent inhibition of the sample compared to the negative control. Each measurement was performed in triplicate, and results are expressed as mean ± standard deviation. The IC50 value represents the concentration (µg mL^−1^) of quercetin or microalgae extracts that produce 50 percent of the enzyme inhibitory capacity compared to the control (without inhibitors) (Equation (7)).

(7) $$\%Inhibition=\frac{V0-V1}{V0}\times 100$$

##### Total Phenolic Content (TPC) 

The determination of total phenolic content (TPC) in the *T. chuii* extracts was carried out using the Folin–Ciocalteu method, as previously described [39]. The TPC results were expressed as milligrams of gallic acid equivalent per gram of dried *T. chuii* extract (mg GAE/g), and all experiments were conducted in triplicate.

#### 3.2.5. Statistical Analysis 

All extraction procedures were carried out in duplicate, and all in vitro analyses were carried out in triplicate. The results are shown as the mean ± standard deviation (SD). In-vitro bioactivity analysis, total phenolic contents, and extraction yield of all the extracted compounds were analyzed by the Pearson test correlation coefficients with 95% confidence. The one-way ANOVA followed by post hoc Tukey’s HSD test at the *p* < 0.05 level was applied to all the results, using software Statistica version 7.1 (Stat-Soft Inc., Tulsa, OK, USA).

#### 3.2.6. Cell Culture Experiments

##### HK-2 and THP-1 Cytotoxicity Assay 

The in vitro toxicity evaluation of the optimized PLE extracts was tested on two different cell culture lines: human proximal tubular epithelial cells (HK-2) and human leukemia monocytic cells (THP-1), with both cell lines being from ATCC^®^ (Rockville, MD, USA). HK-2 and THP-1 cells were grown and maintained as previously described by our group [27,28]. For the cytotoxicity assays on HK-2 cells, cells were seeded in 96-well plates at a density of 5 × 10^3^ cells/well and incubated for 24 h for cell attachment. After this time, cells were treated with different concentrations (1.25 to 40 μg mL^−1^) of extracts (dissolved in ethanol) and incubated for another 24 h. The solvent concentration in no case exceeded 0.4% (*v*/*v*), and medium containing 0.4% (*v*/*v*) ethanol was used as a control. After incubation, the viability of the cells was determined by the using the MTT (3-(4,5-dimethylthiazol-2-yl)-2,5-diphenyltetrazolium bromide) assay as previously described [27,38]. Cell viability was expressed as a percentage (%) and calculated as the ratio of absorbance between extract-treated cells and the ethanol-treated (control) cells. All the experiments were performed in triplicate.

For the cytotoxicity assays on THP-1 cells, cells were seeded in 24-well plates at a density of 5 × 10^5^ cells/well. Monocyte cells were differentiated into macrophages by adding 100 ng mL^−1^ of phorbol 12-myristate 13-acetate (PMA, Sigma-Aldrich) to the cells for 48 h. At the end of this period, the medium was removed, and the cells were washed with PBS, treated with extracts at different concentrations (10, 20, and 40 μg mL^−1^), and incubated for another 24 h. Finally, the viability of cells was assessed by using the same MTT method as described above.

## 4. Conclusions

For the first time, the neuroprotective capacity of *Tetraselmis chuii* has been studied using not only environmentally friendly techniques such as PLE and SFE but also by harnessing the power of green solvents such as CPME and AcOET. PLE allowing for the rapid extraction procedure not only demonstrates the neuroprotective potential of *T. chuii* but also highlights a significant step forward in sustainable and environmentally conscious research practices. By optimizing the PLE conditions, neuroprotective extracts from *T. chuii* were successfully obtained, unlocking their bioactivity to the highest potential. These extracts, obtained using environmentally friendly solvents, are proving to be an excellent source of valuable compounds that can be exploited for a variety of applications. 

Response surface optimization provided two specific conditions of temperature extraction solvent mixture to maximize the bioactive potential of the extracts in only 20 min. Operation at 40 °C and 34.9% AcOEt in CPME yielded high anticholinergic and RNS scavenging capacity (AChE IC50 = 44.26 μg mL^−1^; RNS IC50 = 89.22 μg mL^−1^), whereas operation at 180 °C and 54.1% AcOEt in CPME yielded high anti-inflammatory extracts (LOX IC50 = 11.59 μg mL^−1^). Nevertheless, SFE and the combined process of SFE + PLE provided lower bioactivity. Analysis of these neuroprotective *T. chuii* extracts revealed their composition. They are rich in carotenoids such as neoxanthin, violaxanthin, zeaxanthin, and α- and β-carotene, but in a balanced amount with chlorophylls. Those carotenoids are known for their antioxidant, anti-inflammatory, and anti-cholinesterase properties. In addition, these extracts are also rich in phytosterols and PUFAs, particularly omega-3 fatty acids such as EPA and ALA, further enhancing their therapeutic potential. To evaluate the safety and efficacy of these extracts, in vitro cytotoxicity tests were performed on various cell models. The results were very promising as the extracts showed excellent biocompatibility. In fact, they showed no signs of toxicity in the HK2 and THP-1 cell lines, even at concentrations as high as 40 μg mL^−1^. These results pave the way for future in vivo studies and provide a solid basis for exploring the numerous applications of these green PLE extracts.

To sum up, the use of a green extraction method using CPME and AcOET solvents enabled the discovery of neuroprotective compounds in *Tetraselmis chuii*. This environmentally friendly approach not only highlights the importance of sustainable research practices but also demonstrates the immense potential of green biobased solvents in the extraction of bioactive compounds. The results of this study open new avenues for the use of these environmentally friendly extracts, while further in vivo investigations will fully validate their neuroprotective potential and elucidate the underlying mechanisms responsible for their remarkable effects.

## Figures and Tables

**Table 1:** Matrix of the experimental design for the optimization of *T. chuii* pressurized liquid extraction (PLE) conditions. Factorial experimental design factor levels between parentheses for temperature and solvent composition. Results are expressed as mean ± sd.

Extraction Conditions			Response Variables					
Run	Temperature (°C)	Ethyl Acetate in CPME (%)	TPC	ORAC	RNS	AChE	LOX	Yield (%)
			(mg GAE/g ^§^)	(IC50 µg·mL^−1^)	(IC50 µg·mL^−1^)	(IC50 µg·mL^−1^)	(IC50 µg·mL^−1^)	
1	40 (−1)	0 (−1)	593.94 ± 2.6 ^d^	5.35 ± 0.1 ^e^	79.22 ± 14.5 ^de^	41.11 ± 4.5 ^c^	27.04 ± 2.6 ^c^	3.58 ± 0.2 ^ef^
2	40 (−1)	50 (0)	593.94 ± 2.0 ^d^	6.77 ± 0.2 ^bc^	77.04 ± 10.5 ^e^	52.95 ± 3.4 ^c^	29.53 ± 1.7 ^c^	3.81 ± 0.2 ^e^
3	40 (−1)	100 (1)	628.12 ± 3.7 ^b^	6.79 ± 0.1 ^bc^	86.97 ± 12.3 ^de^	41.21 ± 2.2 ^c^	38.86 ± 4.1 ^b^	3.06 ± 0.2 ^f^
4	110 (0)	0 (−1)	536.14 ± 1.2 ^h^	7.91 ± 0.3 ^a^	91.79± 10.8 ^de^	111.84 ± 1.0 ^ab^	17.91 ± 1.0 ^d^	5.77 ± 0.4 ^c^
5 *	110 (0)	50 (0)	557.01 ± 1.2 ^f^	4.99 ± 0.3 ^ef^	94.18 ± 8.3 ^de^	98.32 ± 10.0 ^b^	16.99 ± 1.4 ^d^	4.79 ± 0.2 ^d^
6 *	110 (0)	50 (0)	562.09 ± 2.1 ^f^	6.18 ± 0.5 ^cd^	88.29 ± 4.7 ^de^	105.37 ± 9.2 ^ab^	13.43 ± 0.4 ^de^	5.94 ± 0.4 ^c^
7 *	110 (0)	50 (0)	602.51 ± 2.5 ^c^	4.24 ± 0.3 ^fg^	76.52 ± 8.9 ^e^	101.34 ± 9.7 ^ab^	18.06 ± 0.8 ^d^	5.87 ± 0.3 ^c^
8 *	110 (0)	50 (0)	550.32 ± 1.2 ^g^	7.12 ± 0.4 ^ab^	89.72 ± 11.5 ^de^	117.47 ± 14.1 ^ab^	11.44 ± 1.2e	5.91 ± 0.3 ^c^
9	110 (0)	100 (1)	579.49 ± 1.2 ^e^	6.90 ± 0.2 ^bc^	98.71 ± 12.6 ^de^	95.06 ± 10.2 ^b^	24.35 ± 2.3 ^c^	5.89 ± 0.3 ^c^
10	180 (1)	0 (−1)	584.31 ± 2.6 ^e^	4.38 ± 0.1 ^fg^	144.72 ± 8.5 ^b^	126.85 ± 10.3 ^a^	11.44± 1.2 ^e^	11.43 ± 0.3 ^a^
11	180 (1)	50 (0)	644.52 ± 2.8 ^a^	3.97 ± 0.5 ^gh^	132.82 ± 11.2 ^bc^	108.74 ± 16.7 ^ab^	10.64 ± 0.5 ^e^	11.37 ± 0.2 ^a^
12	180 (1)	100 (1)	622.57 ± 2.5 ^b^	4.70 ± 0.2 ^efg^	108.97 ± 9.7 ^cd^	128.30 ± 9.6 ^a^	13.31 ± 0.7 ^de^	7.84 ± 0.7 ^b^
		Galantamine				1.40 ± 0.2 ^d^		
		Quercetin					119.20 ± 1.5 ^a^	
		Ascorbic Acid		3.26 ± 0.20 ^h^	990.11 ± 12.6 ^a^			

Superindex meaning: §; GAE: (gallic acid equivalents), *: experimental design center points, IC50: concentration that inhibit 50% the activity, AChE: acetylcholinesterase, LOX: lipoxygenase, ORAC: oxygen radical absorbance capacity, RNS: reactive nitrogen species, TPC: total phenolic compounds. Different superscript letters in the same column indicate significant differences among samples (*p* < 0.05).

**Table 2:** Optimum pressurized liquid extraction (PLE) conditions for *Tetraselmis chuii* and responses predicted by the statistical model and those obtained experimentally. Results are expressed as mean ± sd of three independent experiments.

	Extraction Conditions			Response Variables					
		Temp. (°C)	Ethyl Acetate in CPME (%)	ORAC *	RNS *	AchE *	LOX *	Yield (%)	BuChE *
				(IC50 µg·mL^−1^)	(IC50 µg·mL^−1^)	(IC50 µg·mL^−1^)	(IC50 µg·mL^−1^)		(IC50 µg·mL^−1^)
Optimum 1	Predicted	40	35	-	76.5	47.5	-	-	-
	Experimental	40	35	9.7 ± 0.9	89.2 ± 8.6	44.3 ± 3.4	22.6 ± 3.0	3.9 ± 0.5	48.8 ± 7.0
	% RSD			9.5	9.7	7.8	13.1	12.4	14.3
Optimum 2	Predicted	180	55	3.7	-	-	9	10.4	-
	Experimental	180	55	3.3 ± 0.4	120.5 ± 12.9	82.9 ± 1.9	11.6 ± 1.4	11.48 ± 1.37	65.7 ± 2.4
	% RSD			11.6	10.7	2.2	11.9	11.9	3.6

* ORAC: oxygen radical absorbance capacity, RNS: reactive nitrogen species, AChE: acetylcholinesterase, LOX: lipoxygenase, BuChE: butirilcholinesterase.

**Table 3:** Bioactivities obtained in *Tetraselmis chuii* supercritical CO_2_ extracts.

Extraction Conditions			Response Variables			
Density (kg/m^3^)	Temp. (°C)	Pressure (MPa)	ORAC (IC50 µg·mL^−1^)	AchE (IC50 µg·mL^−1^)	LOX (IC50 µg·mL^−1^)	Yield (%)
780.3	40	15	9.16 ± 1.08	121.26 ± 2.96	45.61 ± 4.64	4.14 ± 0.21
934.9	40	35	21.66 ± 3.64	144.82 ± 5.97	39.56 ± 2.24	2.99 ± 0.09

ORAC: oxygen radical absorbance capacity, AChE: acetylcholinesterase, LOX: lipoxygenase.

**Table 4:** Identified compounds in optimal *Tetraselmis chuii* extracts (see extraction conditions for OP1 and OP2 in Table 2) by GC-QTOF-MS using the trimethylsilyl derivatization procedure.

Peak No	Ret. Time (min)	Ret. Index	Family	Tentative Identification	Match Factor	Formula	Main Fragments (*m*/*z*)	OP-1 (%)	OP-2 (%)
1	11.369	1453	FA (C10:0)	Decanoic acid	75	C10H20O2	229.1609, 117.0358	0.2	0.2
2	13.696	1651	FA (C12:0)	Dodecanoic acid	82	C12H24O2	257.1924, 117.0358	0.2	0.2
3	15.704	1837	Terpene	Neophytadiene	89	C20H38	123.1175, 95.0864	10.02	8.1
4	15.815	1845	FA (C14:0)	Tetradecanoic acid	89	C14H28O2	285.2250, 117.0358	1.2	1.2
5	16.442	1944	FA (C15:0)	Pentadecanoic acid	81	C15H30O2	299.2395, 117.0361	0.2	0.2
6	16.844	-	PUFA (C16:4n-3)	Methyl 4,7,10,13-hexadecatetraenoate	80	C17H26O2	117.0361, 91.0532	0.2	0.1
7	17.237	1979	FA (C16:0)	Hexadecanoic acid, ethyl ester	75	C18H36O2	241.2153, 157.1203	0.1	0.1
8	17.511	1995	MUFA (C16:1n-7)	(E)-9-Hexadecenoic acid	94	C16H30O2	311.2407, 129.0372	2.1	2.5
9	17.565	1995	MUFA (C16:1n-7)	(Z)-9-Hexadecenoic acid	96	C16H30O2	311.2407, 129.0372	1.2	1.2
10	17.753	2042	FA (C16:0)	Hexadecanoic acid	95	C16H32O2	313.2604, 117.0401	12.9	15.8
11	18.866	2158	MUFA (C18:1n-9)	(Z)-9-Octadecenoic acid, ethyl ester	87	C20H38O2	264.2439, 222.2313	3.4	3.1
12	18.964	2180	Terpene	Phytol	96	C20H40O	143.0900, 123.1166	2.9	3.6
16	19.272	2201	PUFA (C18:2n-6)	(Z,Z)-9,12-Octadecadienoic acid	96	C18H32O2	337.2576, 129.0371	2.8	2.5
17	19.322	2208	MUFA (C18:1n-9)	(Z)-Octadec-9-enoic acid	95	C18H34O2	339.2763, 129.0391	17.1	16.8
18	19.339	2218	PUFA (C18:3n-3)	(Z,Z,Z)-9,12,15-Octadecatrienoic acid	93	C18H30O2	335.2424, 129.0388	12.1	11.0
19	19.533	2239	FA (C18:0)	Octadecanoic acid	86	C18H36O2	341.2875, 129.0388	0.7	0.9
20	20.003	2326	MUFA (C19:1n-9)	(E)-10-Nonadecenoic acid	63	C19H36O2	353.3232, 117.0702	0.7	0.4
21	20.143	2326	MUFA (C19:1n-9)	(Z)-10-Nonadecenoic acid	78	C19H36O2	353.3232, 117.0702	0.1	0.1
23	20.623	2359	PUFA (C20:4n-6)	(Z,Z,Z,Z)-5,8,11,14-Eicosatetraenoic acid	94	C20H32O2	117.0470, 91.0535	0.6	0.6
24	20.687	2380	PUFA (C20:5n-3)	(Z,Z,Z,Z,Z)-5,8,11,14,17-Eicosapentaenoic acid	97	C20H30O2	117.0584, 105.0704	11.4	11.9
26	20.978	2426	MUFA (C20:1n-9)	(Z)-11-Eicosenoic acid	93	C20H38O2	367.3044, 117.0370	2.8	3.4
28	24.116	2830	FA (C24:0)	Lignoceric acid	72	C24H48O2	425.3819, 117.0379	0.1	0.2
29	25.444	-	Phytosterol	Campesterol acetate	77	C31H52O2	382.3591, 147.1117	0.2	0.2
30	26.091	3132	Tocopherol	α-Tocopherol acetate	67	C35H60O7	430.3814, 165.0901	0.6	0.3
31	26.269	3226	Tocopherol	α-Tocopherol	79	C29H50O2	502.4224, 237.1305	1.1	1.0
32	26.386	3135	Phytosterol	Cholesterol	72	C27H46O	368.3416, 329.3195	0.1	0.1
33	27.020	-	Phytosterol	Isofucosterol	80	C29H48O	386.3010, 296.2500	2.3	2.2
34	27.063	3250	Phytosterol	Campesterol	94	C28H48O	472.4129, 382.3630	12.5	11.8

Fatty acids (FAs), monounsaturated fatty acids (MUFAs), and polyunsaturated fatty acids (PUFAs).

**Table 5:** Tentatively identified pigments (carotenoids and chlorophyls) in the extracts of *Tetraselmis chuii* by LC-DAD-QTOF-MS/MS.

No	Ret Time (min)	Identification	Molecular Formula	Monoisotopic Mass	Theoretical [M + H]^+^ *m*/*z*	Experimental [M + H]^+^ *m*/*z*	Error (ppm)	λmax (nm)	MS/MS Product Ions
1	0.85	Fucoxanthinol	C40H56O5	616.4128	617.4201	617.4191	1.6	400s, 420, 447	615.4/599.4106/221.1535
2	0.91	Diatoxanthin	C40H54O2	566.4124	567.4197	567.4208	−2.0	400s, 418, 443	549.4085/427.2991/349.2511
3	0.92	Neoxanthin	C40H56O4	604.178	601.4251	601.4266	−2.5	420s, 438, 464	583.4123/565.4004/491.3454
4	0.94	Diadinoxanthin	C40H54O3	582.4073	583.4146	583.4103	7.3	420s, 440, 465	565.4029/547.3991/491.3521
5	1.06	Violaxanthin	C40H56O4	600.4178	601.4251	601.4258	−1.2	400, 420, 448	583.4087/565.3971/491.3425
6	1.19	Prasinoxanthin	C40H56O4	600.4178	601.4251	601.4235	2.6	380, 400, 425	583.4377/565.4224/335.2581
7	1.40	Zeaxanthin/lutein	C40H56O2	568.4280	569.4353	569.4337	2.8	420s, 445, 475	551.4208/489.3705/431.3281
8	1.40	Crocoxanthin	C40H54O	550.4175	551.4213	551.4229	−2.9	420s, 445, 475	533.4096/495.3582/429.3073
9	1.45	Antheraxanthin	C40H56O3	584.4229	585.4432	585.445	−3.1	426, 448, 474	567.4369/493.4007/384.3338
10	1.80	Echinenone I	C40H54O2	566.4124	567.4197	567.4158	6.8	455	549.4025/475.3372/429.3101
11	2.19	Echinenone II	C40H54O2	566.4124	567.4197	567.4141	9.8	460	549.4025/429.3101/411.3075
12	2.60	Divinylchlorophyll a	C55H70MgN405	890.5197	891.5270	891.5319	−5.5	422	613.2292/581.1968/553.2077
13	2.62	7-Hydroxychlorophyll a	C55H72MgN4O6	908.5302	909.5386	909.5311	8.2	422	631.2385/558.2091
14	2.64	Chlorophyll a	C55H72MgN4O5	892.5353	893.5426	893.5299	14.2	422	603.4616/429.3718
15	3.09	Chlorophyll b	C55H70MgN4O6	906.5145	907.5218	907.5199	2.1	420	629.2225/889.6502/601.2252
16	4.66	Pyropheophytin b	C53H70N4O4	826.5397	827.5481	827.5437	5.3	400	549.2468/503.2394
17	5.72	Pheophytin a	C55H74N4O5	870.5659	871.5743	871.5723	2.3	410	593.2761/533.2581
19	7.00	α-Carotene	C40H56	536.4382	537.4455	537.4460	−1.0	418, 447, 474	457.3877/441.3535/414.329
20	7.25	β-Carotene	C40H56	536.4382	537.4455	537.4454	0.1	430s, 452,478	457.3826/413.3202

## Data Availability

Data contained within the article.

## Supplementary Materials

The following supporting information can be downloaded at https://www.mdpi.com/article/10.3390/ijms25073897/s1.
